# Amyloid-Beta Induced Changes in Vesicular Transport of BDNF in Hippocampal Neurons

**DOI:** 10.1155/2016/4145708

**Published:** 2016-01-10

**Authors:** Bianca Seifert, Robert Eckenstaler, Raik Rönicke, Julia Leschik, Beat Lutz, Klaus Reymann, Volkmar Lessmann, Tanja Brigadski

**Affiliations:** ^1^Institute of Physiology, Medical Faculty, Otto-von-Guericke-University, 39120 Magdeburg, Germany; ^2^Institute of Clinical Chemistry and Pathobiochemistry, Medical Faculty, Otto-von-Guericke-University, 39120 Magdeburg, Germany; ^3^German Center for Neurodegenerative Diseases (DZNE), 39120 Magdeburg, Germany; ^4^Institute of Physiological Chemistry, University Medical Center of the Johannes Gutenberg University, 55128 Mainz, Germany; ^5^Center of Behavioral Brain Sciences (CBBS), 39106 Magdeburg, Germany

## Abstract

The neurotrophin brain derived neurotrophic factor (BDNF) is an important growth factor in the CNS. Deficits in transport of this secretory protein could underlie neurodegenerative diseases. Investigation of disease-related changes in BDNF transport might provide insights into the cellular mechanism underlying, for example, Alzheimer's disease (AD). To analyze the role of BDNF transport in AD, live cell imaging of fluorescently labeled BDNF was performed in hippocampal neurons of different AD model systems. BDNF and APP colocalized with low incidence in vesicular structures. Anterograde as well as retrograde transport of BDNF vesicles was reduced and these effects were mediated by factors released from hippocampal neurons into the extracellular medium. Transport of BDNF was altered at a very early time point after onset of human APP expression or after acute amyloid-beta(1-42) treatment, while the activity-dependent release of BDNF remained unaffected. Taken together, extracellular cleavage products of APP induced rapid changes in anterograde and retrograde transport of BDNF-containing vesicles while release of BDNF was unaffected by transgenic expression of mutated APP. These early transport deficits might lead to permanently impaired brain functions in the adult brain.

## 1. Introduction 

The neurotrophin BDNF is an important growth factor supporting the function of the developing and the adult central nervous system. Depending on cell type and expression pattern BDNF coordinates a multitude of biological functions, like growth of neurites, synaptogenesis, and neuroprotection [1–5]. A lack of BDNF has long-range consequences on cellular as well as on systemic level and therefore could underlie different neurodegenerative diseases [6–8]. Deficits in BDNF transport and thus a lack of BDNF support in the respective target area could initiate neurodegenerative processes in the brain [8–12]. Although many previous studies addressed the neuroprotective signalling of neurotrophins, the regulation of neurotrophic support by the different transport processes of BDNF-containing vesicles is not well understood.

So far, the transport of BDNF was investigated by immunohistochemical staining, radioimmunoassay, live cell imaging of fluorescently labeled BDNF, or quantum-dot labelled BDNF [13–15]. The injection of radioactively labeled recombinant BDNF in different brain regions uncovered an extensive anterograde and retrograde transport of BDNF in the central nervous system [13, 16–18]. Live cell imaging of neuronal cultures expressing fluorescently labeled BDNF revealed a velocity of BDNF-containing vesicles of 0.1–1.4 *µ*m/s [14, 19–21]. In both directions, the transport of BDNF-containing vesicles is dependent on the dynactin-motor complex [14, 19, 20]. In transgenic mouse models of Alzheimer's disease, an impaired retrograde transport of endocytosed BDNF was described by Poon and colleagues. They observed a deficit in retrograde transport of endocytosed BDNF/TrkB-signalling endosomes which was associated with amyloid-beta induced downregulation of ubiquitin C-terminal hydrolase [22, 23]. A similar influence of amyloid precursor protein (APP) or cleavage products of APP on activity-dependent secretion of BDNF has not yet been described.

In this study, we demonstrate a fast action (within 5 min) of amyloid-beta(1-42) on the transport of BDNF-containing vesicles. BDNF and APP colocalized with low incidence in vesicular structures and the degree of colocalization increased with higher APP expression level in a cell. However, deficits in anterograde as well as retrograde BDNF transport were not correlated with APP expression at the single cell level but were rather induced by soluble factors released from hippocampal neurons into the extracellular medium. Furthermore, short application of amyloid-beta(1-42) but not amyloid-beta(3(pE)-42) induced a reduction in average speed of retrogradely directed vesicles. Anterogradely directed vesicles were affected by transgenic expression of APP as well as by amyloid-beta treatment at later time points of culturing. Nevertheless, activity-dependent release of BDNF was not affected by any of these manipulations affecting the transport of BDNF vesicles.

## 2. Materials and Methods

### 2.1. Cell Culture

All experiments were performed in accordance with the ethical guidelines for the use of animals in experiments and were approved by the local animal care committee (Landesverwaltungsamt Sachsen-Anhalt).

Hippocampal neurons of C57BL/6 mice were isolated at postnatal day 2 and prepared as described previously [4, 24] with minor modifications: primary postnatal (P2) neocortical astrocytes were isolated and cultured for 2-3 weeks in BME medium, containing 10% FCS until being expanded to confluence. Astrocytes were passaged and seeded on glass coverslips at a density of 80 000 cells per 3.5 cm dish in BME/10% FCS to yield astrocyte islands of 100–300 *µ*m in diameter after 7–14 days* in vitro* (DIV). *β*-D-Arabinofuranosylcytosine (3 *µ*M) was added 4 d after seeding of astrocytes to avoid further growth of astrocyte islands. Dissociated postnatal (P2) hippocampal neurons were plated in BME/10% FCS at a density of 1–10 neurons per astrocyte island onto the coverslips. After 20 h, the plating medium was exchanged to serum-free medium (Neurobasal with 2% B27 supplement; Invitrogen, San Diego, CA).

Hippocampal neurons from 5xFAD mice were isolated at postnatal day 2 and plated at a density of 70 000 cells per cm^2^ on a polyornithine-coated cover slip. After 20 h, the plating medium was exchanged to serum-free medium (Neurobasal with 2% B27 supplement; Invitrogen, San Diego, CA).

### 2.2. Transfection

Hippocampal cultures were transfected with expression vectors coding for BDNF-mCherry, HA tagged proBDNF-GFP, APP-YFP, APPmCherry, or GFP, respectively [25, 26], using the Ca^2+^-phosphate precipitation method described previously [25] with minor modifications: hippocampal cultures of C57BL/6 mice were transfected at 9 DIV and hippocampal cultures of transgenic 5xFAD mice were transfected at DIV 10 with the respective expression plasmids. Cells were used for experiments 1–3 days after transfection.

### 2.3. Live-Cell Imaging and Image Analysis

Coverslips were transferred into Petriperm dishes (Greiner Bio-One, Frickenhausen, Germany) containing HEPES buffered saline (20 mM HEPES, 4 mM KCl, 100 mM NaCl, 1 mM Na_2_HPO_4_, 4 mM CaCl_2_, 10 mM glucose, 10 *µ*M glycin, and 1 mM MgCl_2_; pH 7.4) and imaged with an inverted epifluorescence microscope (IX70, Olympus, Hamburg Germany) using high aperture oil immersion objectives (40x, n.a.: 1.0) and a CCD camera (Sensys 1401E, Photometrics) as described previously [27] with minor modifications: cells were kept at a constant temperature of 30°C using a plate warmer (Minitüb, Tiefenback, Germany). Time-lapse images were acquired at a frequency of 2 Hz with an exposure time of 300 ms. 1-minute time-lapse recordings were analyzed with MetaMorph software (Molecular Devices, Downington, PA, USA). Dynamics of BDNF-containing vesicles were analyzed in individual thin neurites by manual tracking using the “track points” function of MetaMorph. Vesicles were tracked for 1 min or until they disappeared out of view. BDNF-containing vesicles were considered as immobile when the percentage of motion during observation time was lower than 15% or the minimal distance of movement during observation time was below 8 *µ*m. Vesicles which showed diffusing behavior estimated by the ratio of displacement and distance (>4) were excluded. Otherwise the vesicles were defined as mobile.

Colocalization of proteins was examined with a confocal laser scanning microscope (LSM 780, Zeiss, Jena, Germany) using a 20x water immersion objective (W Plan-ACHROMAT, numerical aperture: 1.0). Images were acquired and analyzed with the ZEN 2010 software. Weighted colocalization coefficient was calculated by the Zen software.

### 2.4. Immunohistochemistry

Cells were fixed with 4% paraformaldehyde in PBS (pH 7.4) for 20 min and permeabilized with 0.3% Triton X-100 in PBS. Unspecific binding was inhibited by incubation with blocking solution (1x PBS, 10% BSA and 0.1% Triton X-100) for 15 min. Incubation with primary and secondary antibodies was done for 2 h in PBS containing 1% BSA and 0,1% Triton X-100. Cells were fixed on a glass slide with Immumount (Thermo Scientific, Waltham, MA, USA) and stored at 4°C until analysis. All steps were performed at room temperature. Primary antibodies used were anti-APP (rabbit, 1 : 1000, Upstate, Millipore Corporation, USA) and anti-HA (mouse, 1 : 1000, Covance, New Jersey, USA). Secondary antibodies used were Cy3 coupled anti-rabbit IgG (1 : 1000, Dianova, Hamburg, Germany) and Alexa Fluor 350 coupled anti-mouse IgG (1 : 1000, Moleculare Probes, Life Technologies, Carlsbad, California, USA). Colocalization analysis was performed by MetaMorph software (Molecular Devices, Downingtown, PA).

### 2.5. Preparation of Amyloid-Beta Oligomers

Amyloid-beta oligomers were generated as described in [28]. Briefly, the lyophilized peptides (amyloid-beta(1-42), MoBiTec) were dissolved in 1,1,1,3,3,3-hexafluoro-2-propanol (HFIP; Sigma, St. Louis, MO) to a concentration of 1 mM. The solution was aliquoted, HFIP was evaporated, and the peptide film was stored at −80°C. Twenty-four hours before use, amyloid-beta peptides were dissolved under sterile conditions in dimethylsulfoxide (DMSO; Sigma; 100 *μ*M) and sonicated. Amyloid-beta oligomers were obtained by diluting the stock solution to 5 *μ*M in NB/B27 and incubating at 4°C for 24 h. In previous experiments, the quality of the oligomer preparation was controlled by negative stain electron microscopy and with sodiumdodecylsulfate-polyacrylamidgelelectrophoresis (SDS-PAGE) [29]. Early neuronal dysfunction by amyloid-*β* oligomers depends on activation of NR2B-containing NMDA receptors. We already investigated this oligomer preparation for detrimental effects on neuronal function and neuronal structural integration. Using primary neuronal cell culture and hippocampal slices from rat and mouse, we found that administration of submicromolar concentrations of A*β* oligomers readily impairs long-term potentiation, reduces baseline synaptic transmission, decreases neuronal spontaneous network activity, and induces retraction of synaptic contacts [29].

Short-term treatment of hippocampal neurons with 500 nM amyloid-beta peptides (dissolved in HBS) was performed in the recording chamber for 5–30 min. Long-term incubation of hippocampal neurons with 500 nM amyloid-beta peptides was performed in NB/B27 culture medium for 24 h in the cell culture incubator.

### 2.6. Release Measurements

Primary hippocampal neurons were transfected with BDNF-GFP expressing plasmid at 10 DIV and imaged essentially as described previously [24]: three days after transfection, cover slips with hippocampal neurons were transferred into a bath chamber (Luigs & Neumann) and inspected with an upright fluorescence microscope (Olympus BX51W) using a 60x water immersion objective (LUMFI, Olympus, Hamburg, Germany, NA: 1.1). Image capture was performed using a cooled CCD camera (CoolSnap HQ^2^, Photometrics, Huntington Beach, CA, 14 bit dynamic range), controlled by VisiView software (Visitron Systems, Puchheim, Germany). The exposure times for time-lapse recordings (between 0.3 and 1.5 sec) were adjusted for each cell such that vesicles were clearly distinguishable from the background without driving the CCD chip into saturation. BDNF-GFP release was stimulated by applying 50 mM KCl containing HEPES buffer with a local perfusion system. To estimate the incidence of vesicle fusion, 0.3 mM bromphenol blue (BPB; compare [30]) containing HEPES buffer was superfused after the release experiment. Analysis of images was performed by MetaMorph software (Molecular Devices, Downingtown, PA). In brief, single vesicles were selected to measure the average fluorescence kinetics of single cells. Background fluorescence intensities were subtracted for vesicle and the average intensity was normalized to the time point before fusion event. A monoexponential extrapolation of the photo bleaching observed during baseline recordings was applied to correct the normalized fluorescence data [30]. For analysis of release amplitude maximum fluorescence intensity of each single secretory granule after fusion event (time point = 0) was set to 100% and relative fluorescence decrease 300 s after fusion was analyzed. The percentage of fusion events was calculated by the fraction of vesicles disappearing during BPB application after release experiment. This fraction of vesicles could be also detected by the change in fluorescence intensity after fusion event.

### 2.7. Statistics

Statistical analysis was performed using SPSS version 21 software (IBM Corp., Armonk, NY, USA) using either one-way or two-way ANOVA following* post hoc* Tukey's or pairwise multiple comparisons as indicated in the text and figure captions. The level of significance was set at *p* < 0.05.

## 3. Results

### 3.1. Motional Properties of BDNF-Containing Vesicles In Transgenic Mouse Models of Alzheimer's Disease

**Figure 1:**
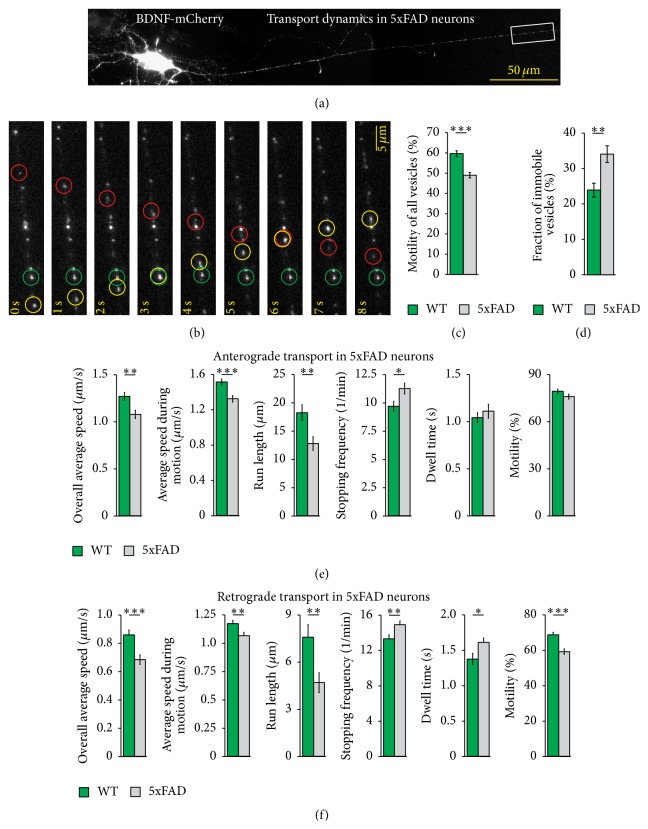
Transport dynamics of BDNF-containing vesicles in hippocampal neurons of 5xFAD mice. Dissociated hippocampal neurons of transgenic mice with five familial Alzheimer's disease mutations (5xFAD) and wild-type littermates were transfected at 10 DIV with fluorescently labeled BDNF. The dynamic behavior of BDNF-containing vesicles was analyzed by live cell imaging. (a) Representative picture of a hippocampal neuron transfected with BDNF-mCherry. (b) Boxed area from (a) at higher magnification and at different time points of time-lapse recording. Red and yellow circles mark BDNF-containing vesicles showing a directional movement. Red circle marks a vesicle which moves in anterograde direction from the soma to distal part of the neurite, while yellow circle marks a vesicle which moves in retrograde direction from distal part of the neurite to the soma of the neuron. Green circle marks a vesicle which was immobile during observation. (c) Bar diagram showing the mean motility of BDNF-containing vesicles in hippocampal neurons of 5xFAD mice compared to wild-type littermates. Note that the motility of BDNF-containing vesicles was significantly reduced in hippocampal neurons from 5xFAD mice compared to wild-type littermates. (d) Bar diagram indicates the percentage of immobile BDNF-containing vesicles. Note that there are significantly more immobile vesicles in neurons of transgenic animals compared to wild-type littermates. (e) Transport characteristics of mobile anterogradely directed BDNF-containing vesicles. Bar diagrams show motional properties of BDNF-containing vesicles as indicated. The overall average speed and the average speed during motion, run length, and stopping frequency of BDNF-containing vesicles were significantly changed in neurons of transgenic animals compared to wild-type littermates. (f) Motional properties of retrogradely directed BDNF-containing vesicles. All motional properties of BDNF-containing vesicles moving in retrograde direction were significantly changed in neurons of transgenic animals compared to wild-type littermates. (^*∗*^ *p* < 0.05, ^*∗∗*^ *p* < 0.01, ^*∗∗∗*^ *p* < 0.001; one-way ANOVA). Error bars represent SEM.

To analyze the role of BDNF transport in Alzheimer's disease, live cell imaging of fluorescently labeled proteins was performed in dissociated hippocampal neurons from an Alzheimer's disease mouse model (5xFAD) [31–33]. This transgenic mouse line was engineered to overexpress A*β*(1-42) [32, 34] by coexpressing amyloid precursor protein (APP) with three familial Alzheimer's disease (FAD) mutations and presenilin 1 (PS1) with two FAD mutations which additionally increases A*β*(1-42) production. Both transgenic proteins are under the control of the Thy1 promoter which drives early postnatal neuron-specific transgenic expression [35, 36]. Dissociated hippocampal cultures derived from these 5xFAD animals and their wild type littermates showed similar cell density and cell survival after 12 days* in vitro *(DIV) (Supplementary Figure  1 in Supplementary Material available online at http://dx.doi.org/10.1155/2016/4145708). These cultures were transfected with BDNF-mCherry at 10 DIV (Figure 1(a)) and transport dynamics of single BDNF-containing vesicles in thin processes with axonal morphology were analyzed by time-lapse video microscopy. During the observation time BDNF-containing vesicles either showed stop-and-go movements or were immobile (Figure 1(b)). Analyzing the motility of BDNF-containing vesicles, as quantified by the percentage of motions during observation, we observed a significant reduction in vesicular motility in 5xFAD derived hippocampal neurons compared to wild-type littermates. While the mean motility of BDNF-containing vesicles was 59.7 ± 1.3% under control conditions, the mean motility of BDNF-containing vesicles was significantly reduced to 49.1 ± 1.3% in 5xFAD transgenic hippocampal neurons (*p* < 1*∗*10^−7^; Figure 1(c)). A similar result was obtained by analyzing the fraction of mobile and immobile vesicles. While 76.0 ± 2.0% of BDNF-containing vesicles displayed a directed movement in wild-type neurons, only 65.8 ± 2.4% of BDNF-containing vesicles were actively transported in 5xFAD transgenic neurons. Accordingly, a larger proportion of vesicles was immobile in these transgenic neurons showing no active transport at all (WT: 24.0 ± 2.0%; 5xFAD: 34.2 ± 2.4% *p* < 0.0013; Figure 1(d)). Altogether, these results indicate a transition of BDNF-containing vesicles from mobile to an immobile state in 5xFAD mice derived neurons.

We next asked whether, in addition to the increased number of immobile vesicles, also motional properties of dynamic vesicles were affected by the transgene expression. Since the vesicle motion was rather characterized by a stop-and-go behavior than by moving at constant speed, different motional properties of BDNF-containing vesicles were analyzed. To this end, we determined the stopping frequency which is defined by the number of stops per unit of time (Supplementary Figure 2). Furthermore, we analyzed the dwell time, expressed by the time the vesicles spent in the same position during one stop. Both stopping frequency and dwell time characterize the stopping phase of a vesicle. During the mobile phase (go), the vesicles move with a certain speed which is described by the average speed of the vesicles during motion, omitting the stops of the vesicles. The covered distance of vesicles between two stops is described by the run length (cf. [37]). The overall average speed of BDNF-containing vesicles which differs from the average speed during motion was defined as the ratio of distance covered to the observation time (cf. [38]) (Supplementary Figure 2). Finally, the percentage of motions occurring during the observation time was determined as a read-out of the motility of one vesicle. While the motility as well as the dwell time was similar for anterogradely directed BDNF-containing vesicles under both conditions (motility: 5xFAD: 75.50 ± 1.57%; Ctrl: 78.91 ± 1.33%; dwell time: 5xFAD: 1.10 ± 0.07 s; Ctrl: 1.04 ± 0.05 s), the stopping frequency of anterogradely directed vesicles was significantly increased in transgenic neurons as compared to wild-type neurons (Figure 1(e)). In addition to the stopping frequency of anterogradely directed vesicles, the run length as well as the average speed during motion was significantly decreased in transgenic neurons resulting in a reduced overall average speed of BDNF-containing vesicles (Figure 1(e)). All motional properties of retrogradely directed BDNF-containing vesicles were significantly changed in transgenic neurons, compared to wild-type neurons (Figure 1(f)). Altogether, the overall average speed of BDNF-containing vesicles was reduced in transgenic neurons due to a lower speed during motion and a higher number of stops during the active transport of BDNF-containing vesicles.

### 3.2. The Deficits in BDNF Transport Are Also Observed upon Acute Overexpression of hAPP but Occur Independently of Intracellular Levels of APP

**Figure 2:**
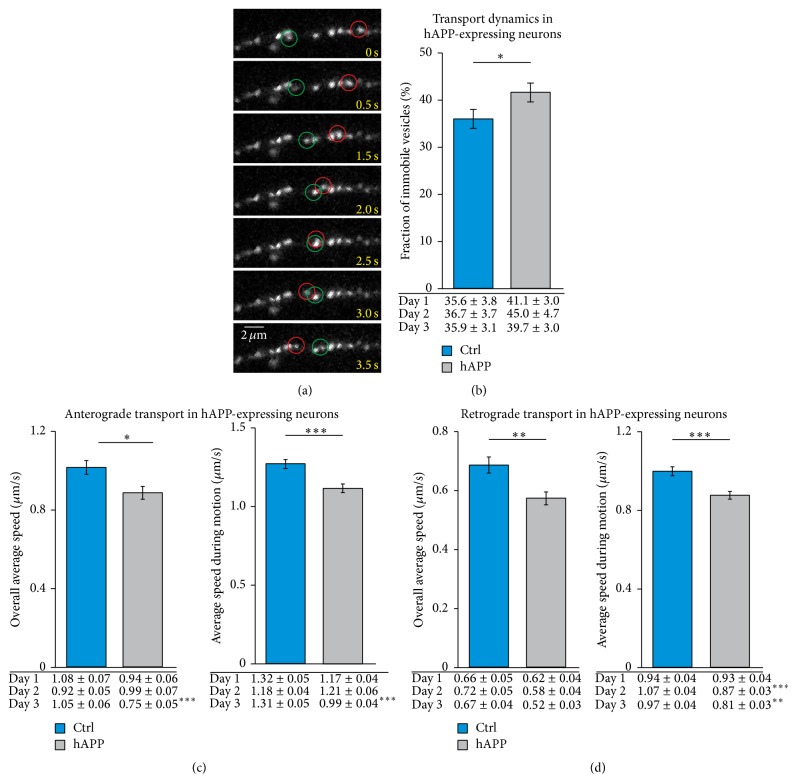
Expression of hAPP reduced vesicular transport of BDNF. Dissociated hippocampal neurons of C57BL/6 mice were cotransfected at 10 DIV with BDNF-mCherry and hAPP-YFP. The dynamic behavior of BDNF-containing vesicles was analyzed by live cell imaging 1–3 days after transfection. (a) Representative picture of BDNF-containing vesicles at different time points of time-lapse recording. Red and green circles mark BDNF-containing vesicles showing a directional movement. (c) Bar diagram indicates the mean percentage of immobile BDNF-containing vesicles in control neurons or in neurons transfected with hAPP during the whole observation period. Tables below bar diagram give the mean value of the incidence of immobile vesicles for day 1, day 2, and day 3. Transient expression of hAPP significantly changed the proportion of immobile vesicles. (d) Transport dynamics of anterogradely directed BDNF-containing vesicles. Bar diagrams show the mean value of the indicated motional properties during the whole observation period. Tables below bar diagrams give the mean value of the indicated motional property for day 1, day 2, and day 3. The overall average speed as well as the average speed during motion was significantly reduced in neurons transfected with hAPP compared to control neurons. While the characteristics of BDNF-containing vesicles were similar in neurons expressing hAPP and control neurons at day 1 after expression of hAPP, the average speed of BDNF-containing vesicles was significantly reduced in neuronal cultures transfected with hAPP at day 3. (e) Transport dynamics of retrogradely directed BDNF-containing vesicles. The motional properties as indicated were significantly changed in neurons transfected with hAPP compared to controls (^*∗*^ *p* < 0.05, ^*∗∗*^ *p* < 0.01, ^*∗∗∗*^ *p* < 0.001; two-way ANOVA followed by pairwise multiple comparison). Error bars represent SEM.

**Figure 3:**
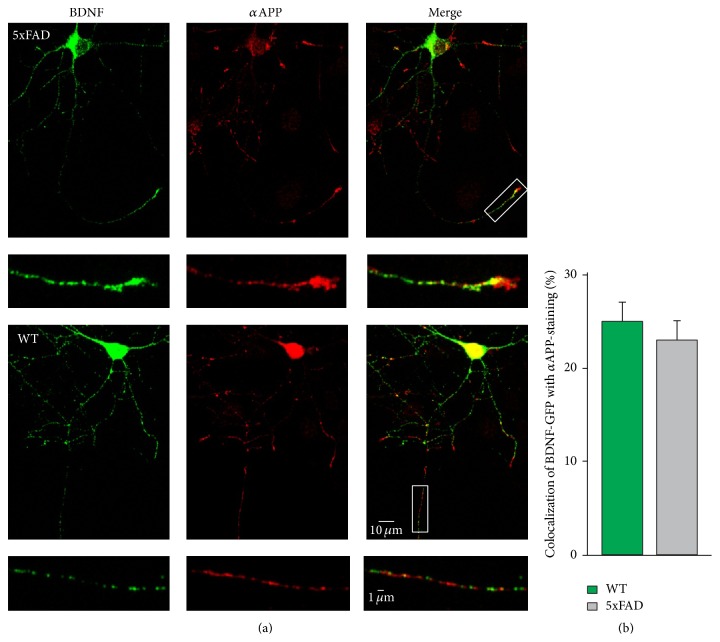
A subpopulation of BDNF-containing vesicles colocalize with APP. (a) Dissociated hippocampal cultures of 5xFAD mice and wild-type littermates were transfected with BDNF-GFP (green) and immunostained with an antibody directed against the N-terminus of endogenous APP (red). Representative pictures showing a transfected and immunostained hippocampal WT neuron (upper part) and 5xFAD neuron (lower part) and higher magnification of boxed areas. Mature BDNF colocalized with APP in vesicular structures. (b) Percentage of *α*APP staining colocalizing with total BDNF-GFP area. Error bars represent SEM.

The Thy1 promoter in 5xFAD mice initiates transgene expression in neuronal cells at postnatal day 6 [35, 36]. To determine how fast the expression of human APP (hAPP) can negatively influence the transport of BDNF-containing vesicles, we cotransfected hippocampal neurons from C57BL/6 animals with BDNF-mCherry and GFP or APP-YFP, respectively, to mimic an early increased expression of hAPP. Again, the number of immobile vesicles was increased in hippocampal neurons transfected with BDNF-mCherry and hAPP-YFP compared to neurons transfected with BDNF-mCherry and GFP as a control (Figure 2(b)). In addition, the transport dynamics of mobile BDNF-containing vesicles were significantly changed in hippocampal neurons expressing hAPP compared to control neurons (Figures 2(b)–2(d), Supplementary Figure 3). Expression of hAPP in hippocampal neurons revealed similar transport deficits for anterograde and retrograde motion as observed in 5xFAD mice-derived neurons. The onset of BDNF transport deficits occurred three days after the initiation of hAPP expression. Since the expression level of APP varied among the cells investigated, we asked whether the magnitude of transport deficits correlated with the cellular content of APP. Therefore, the fluorescence intensity of APP-YFP in the cell body of the investigated cells (which estimates the individual intracellular APP-level) was plotted against the motional properties of the respective individual cell. However, no correlation between the level of APP and transport deficits was obvious (Supplementary Figure 4). Thus, although the extent of transport deficits increases with the duration of APP expression in hippocampal cultures, there was no evidence that higher amounts of cellular APP negatively influence the transport of mobile BDNF-containing vesicles in the same cell. However, the overall intracellular APP expression level does not uncover the involvement of APP or APP-derived molecules in different aspects of cellular functions at the subcellular level. For example, previous studies suggested a function of APP as an adapter protein for cargo transport ([39, 40] but see [41]). Therefore, single APP molecules might directly interact with BDNF-containing vesicles on the subcellular level. Thus, to examine a function of APP as adapter protein for BDNF vesicle transport, we investigated whether both proteins colocalize at the subcellular level. Hippocampal neurons transfected with proBDNF-GFP were immunostained with an antibody directed against the N-terminal part of APP. The mature domain of BDNF colocalized with low frequency with the N-terminal part of endogenous APP in vesicular-like structures (Figure 3(a)). In addition, also the prodomain of BDNF which predominantly colocalizes with the mature domain of BDNF reveals clearly detectable but relatively low levels of colocalization of ProBDNF and APP (Supplementary Figure 5). These results suggest modest localization of BDNF with APP within the same structure in hippocampal neurons. Nevertheless, APP might act as an adapter protein for a subpool of BDNF-containing vesicles. However, the absence of any correlation between the strength of APP expression and the motional properties in individual cells indicated that not the levels of overexpressed hAPP in a given cell were responsible for impairing transport properties, suggesting rather that APP cleavage products in the culture medium are more likely to be the trigger for the impaired transport properties.

### 3.3. A*β* Peptides Induce Transport Deficits on a Short Time Scale

To test whether the disturbance of BDNF transport is mediated by factors released into the extracellular medium, hippocampal neurons cotransfected with BDNF-mCherry and GFP were cultured after transfection together with hippocampal neurons cotransfected with BDNF-mCherry and hAPP-YFP (Supplementary Figure 6). Neurons cotransfected with BDNF-mCherry and GFP which were transferred into a culture dish with neurons also cotransfected with BDNF-mCherry and GFP served as control. Transport dynamics of single BDNF-containing vesicles were analyzed by time-lapse video microscopy. Interestingly, the fraction of immobile vesicles was similar in both conditions (Ctrl in hAPP media: 31.2 ± 2.4%; Ctrl in Ctrl media: 27.8 ± 3.0%; *p* = 0.34, two-way ANOVA). However, the transport deficits of mobile BDNF-containing vesicles observed for control neurons in hAPP medium (Ctrl in hAPP) were comparable to the transport deficits seen after hAPP expression in hippocampal neurons (cf.  Figure 2). The average speed during motion and the overall average speed for anterogradely directed BDNF-containing vesicles were significantly reduced in hippocampal neurons transferred into culture medium of hippocampal neurons transfected with hAPP (Supplementary Figure 6). Thus, an extracellular factor which is produced in hippocampal neurons transfected with hAPP and released into extracellular medium might be responsible for the observed deficits in transport of BDNF (Supplementary Figure 6).

**Figure 4:**
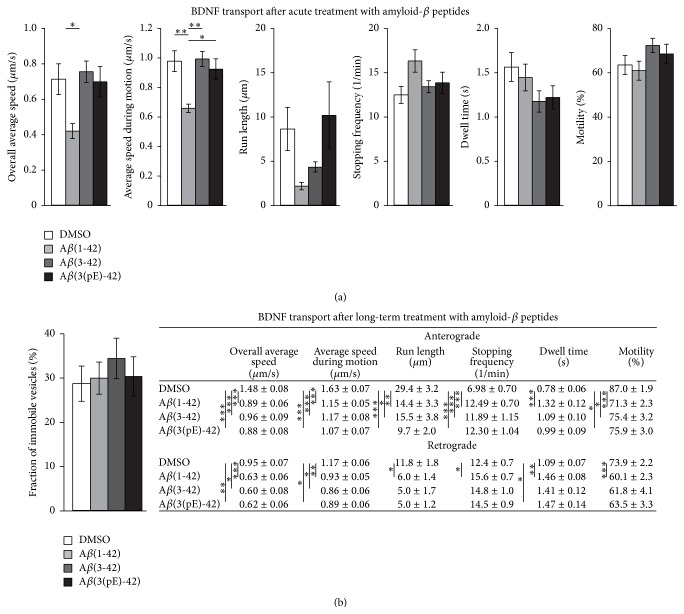
Application of amyloid-beta(1-42) altered BDNF transport with faster onset than observed for other A*β* peptides. Dissociated hippocampal neurons derived from 5xFAD wild-type littermates were transfected at 10 DIV with fluorescently labeled BDNF. The dynamic behavior of BDNF-containing vesicles was analyzed by live cell imaging after (a) acute and long-term (b) treatment with different amyloid-beta peptides. (a) Acute treatment with amyloid-beta(1-42) influenced average speed of retrogradely directed BDNF-containing vesicles. Bar diagrams show the mean values of the indicated motional properties after acute treatment with different amyloid-beta peptides. The average speed during motion was significantly reduced in neurons treated with amyloid-beta(1-42) compared to neurons treated with solvent (0,1% DMSO), amyloid-beta(3-42), or amyloid-beta(3(pE)-42), respectively (^*∗*^ *p* < 0.05, ^*∗∗*^ *p* < 0.01, one-way ANOVA followed by* post hoc* Tukey Test). (b) Long-term treatment with amyloid-beta (24 h) influenced different motional properties of BDNF transport. (left) Bar diagram shows the percentage of immobile BDNF-containing vesicles in hippocampal neurons after treatment with different amyloid-beta peptides. (right) Table shows the mean values for the different motional properties of anterogradely and retrogradely directed BDNF-containing vesicles in hippocampal neurons. Note that all motional properties were significantly changed after 24 h treatment with amyloid-beta(1-42) compared to control conditions. Long-term incubation with amyloid-beta(3-42) or amyloid-beta(3(pE)-42) also significantly influenced some motional properties of BDNF-containing vesicles (^*∗*^ *p* < 0.05, ^*∗∗*^ *p* < 0.01, ^*∗∗∗*^ *p* < 0.001; one-way ANOVA followed by* post hoc* Tukey's test). Error bars represent SEM.

The proteolytic products of APP which are secreted into extracellular space play an important role in the pathogenesis of Alzheimer's disease. One of the best studied and most toxic amyloid-beta peptides is amyloid-beta(1-42) which is a major component of amyloid plaques. Another amyloid species, the N-terminally truncated and modified amyloid-beta(3pE-42), was also described to be upregulated in Alzheimer's disease brains. Both peptides are known for their high toxicity as well as for rapid accumulation in brains of 5xFAD mice [32, 42] and are important players in the pathogenesis of Alzheimer's disease. To analyze whether one of these proteolytic products is responsible for the observed deficits in BDNF transport, hippocampal neurons transfected with BDNF-mCherry were treated with different amyloid-beta oligomers. Amyloid-beta treatment was performed either acutely (5–30 min) or for a time period of 24 h. Acute treatment with amyloid-beta(1-42) but not the N-terminally truncated amyloid-beta(3-42) or the truncated and modified amyloid-beta(3(pE)-42) significantly decreased the average speed of retrogradely directed BDNF-containing vesicles (Figure 4(a)). All other motional properties were not significantly altered after this short time period of amyloid-beta treatment (Supplementary Figure 6). However, these motional properties were significantly altered after incubation with amyloid-beta(1-42) for 24 h (Figure 4(b)). Likewise, BDNF transport dynamics were also affected by long-term incubation with amyloid-beta(3-42) or amyloid-beta(3pE-42). Nevertheless, although all motional properties of mobile BDNF-containing vesicles were significantly changed by amyloid-beta(1-42), there was no change in the number of immobile vesicles (Figure 4(b)) which suggest a possible role of intracellular APP on detachment of moving vesicles from microtubules. Taken together, these data reveal a pronounced slowing for most aspects of BDNF-vesicle movement by the different soluble amyloid-beta peptides tested with earliest onset for amyloid-beta(1-42) induced effects on average speed of BDNF-containing vesicles.

### 3.4. Release of BDNF Is Not Affected in Neurons from 5xFAD Mice

**Figure 5:**
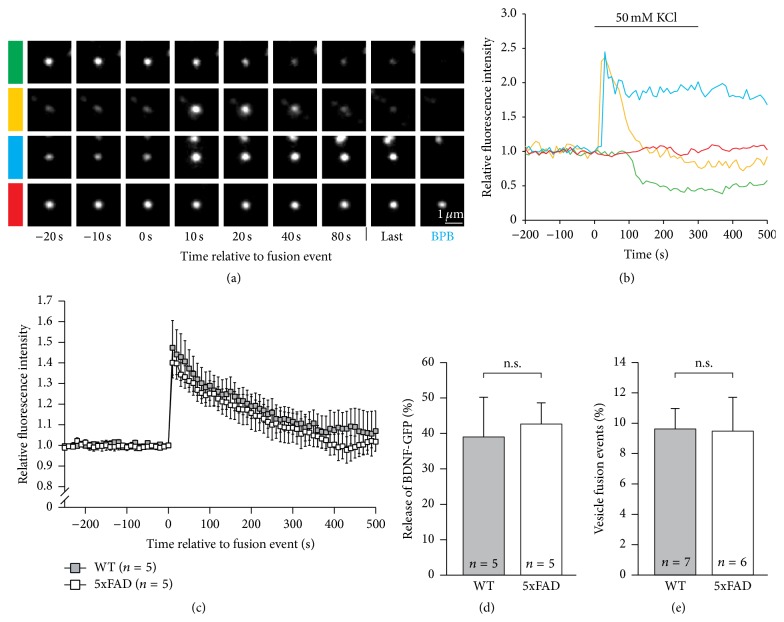
Release of BDNF was not affected after expression of mutated hAPP. Dissociated hippocampal neurons from 5xFAD mice and wild-type littermates, respectively, were transfected at 10 DIV with a BDNF-GFP plasmid. Depolarization-induced release of BDNF-GFP as well as the incidence of fusion event of BDNF-containing vesicles was analyzed by live cell imaging. (a) Representative single vesicles of a hippocampal neuron transfected with BDNF-GFP showing fusion events as indicated by the change in fluorescence intensity (green, yellow, and blue) compared to a single vesicle showing no fusion pore opening (red). For most events, BDNF-GFP fluorescence increased due to the neutralisation of the intragranular pH after fusion. BDNF release was observed as decrease in fluorescence intensity after fusion event. At the end of the release measurement, neurons were superfused with BPB (0.3 mM) to differentiate between vesicles in an open or closed state at this time point (scale bar = 1 *µ*m). (b) Graph shows time course of fluorescence intensity of color coded vesicles shown in (a). Change in fluorescence intensity indicates neutralisation of intravesicular pH after fusion pore opening of BDNF-GFP-containing vesicles (fluorescence increase) and depolarization-induced release of BDNF-GFP (fluorescence decrease), respectively. (c) Average time course of GFP-fluorescence intensity of single hippocampal neurons. Note the absence of any difference in release efficiency or time course between neurons from 5xFAD animals and wild-type littermates. (d) Quantification of the fluorescence decrease after fusion event. The maximal fluorescence intensity after fusion was set to 100% and the remaining fluorescence intensity 300 s after fusion pore opening was analyzed. BDNF release amplitude was unaltered in hippocampal neurons derived from 5xFAD mice. (e) Quantification of depolarization-induced fusion events. Bar diagram shows the percentage of fusion events of vesicles in hippocampal neurons from 5xFAD animals and wild-type littermates (n.s. = no significant difference; one-way ANOVA). Error bars represent SEM.

Reduced transport efficiency of BDNF vesicles could eventually decrease the number of BDNF-containing vesicles ready for exocytosis. Therefore, we analyzed whether activity-dependent release of BDNF was affected by transgenic expression of mutated hAPP. Hippocampal neurons from 5xFAD mice were transfected with BDNF-GFP and depolarized by local superfusion with 50 mM potassium in extracellular solution [24]. The depolarization-induced release of BDNF was analyzed by monitoring GFP fluorescence intensity of BDNF-GFP-containing vesicles using time-lapse video microscopy (all experimental conditions as in Figure 1). Changes in fluorescence intensity became evident 10–50 s after onset of depolarization (Figure 5(b)). Increase in fluorescence intensity due to neutralisation of intravesicular pH after fusion pore opening of BDNF-GFP containing vesicles was followed by decrease in fluorescence intensity indicating depolarization-induced release of BDNF (Figures 5(b)-5(c)). Increase in fluorescence intensity as well as decrease in fluorescence intensity after fusion event was similar in both groups. Furthermore, the density of BDNF-GFP containing vesicles (data not shown) as well as number of vesicular fusion events was similar under both conditions (Figures 5(d)-5(e)). Therefore, neither percentage of fusion events nor release of BDNF from single vesicles was changed in hippocampal neurons derived from 5xFAD mice. Thus, although the transport of BDNF vesicles was affected very early after transgenic expression of hAPP, activity-dependent release of BDNF was not affected.

These results suggest that APP plays a role in transport of BDNF rather than in exocytosis of BDNF-containing vesicles.

## 4. Discussion 

In the present study, we show that different manipulations increasing either APP protein expression or the level of APP-derived molecules in the extracellular medium disturbed retrograde and anterograde transport of the neurotrophin BDNF. Thus, transgenic overexpression of mutated hAPP in 5xFAD mice, plasmid-driven overexpression of hAPP in hippocampal cultures, or extracellular application of amyloid-beta peptides all decreased anterograde as well as retrograde transport of BDNF vesicles. The effects of soluble A*β* peptides could be shown to occur on a fast time scale (within 30 min). Although, the proteins APP and BDNF colocalize with low probability in vesicular structures, the deficits in BDNF transport were not correlated with APP levels at the single cell-level but were mediated by factors released from hippocampal neurons into the extracellular medium. Retrograde transport was affected prior to the anterograde direction, suggesting different mechanisms being responsible for the direction specific deficits. Despite reduced transport efficiency, activity-dependent release of BDNF was not affected by transgenic expression.

The neurotrophic factor BDNF is a very important growth factor for the development of synapses and the survival of neurons [2, 3, 5, 43–45]. A lack of BDNF in the target area can lead to neuronal loss [6–8]. In addition to a reduced level of BDNF, a deficit in transport of BDNF can also result in a lack of neurotrophic support in the respective target areas [6, 8, 9, 11]. BDNF is stored in secretory vesicles which are sorted to either the constitutive or the activity-dependent pathway of secretion [24, 46, 47]. Proteolytic cleavage events of the proBDNF precursor can take place either in intracellular compartments or in the extracellular space, following secretion [25, 47–49]. Our results confirm that the prodomain of BDNF and mature BDNF colocalize very well in hippocampal neurons (Supplementary Figure 5). Nevertheless, colocalization of both domains cannot rule out proteolytic processing which takes place inside these secretory vesicles. The protein APP has also been shown to undergo proteolytical processing by different secretases and is sorted into secretory granules [50–55]. Our results now reveal for the first time that BDNF and APP can colocalize, albeit with low abundance, in vesicular structures of hippocampal neurons (Figure 3). This observation is consistent with previous reports indicating kinesin-1-dependent transport of both cargoes in neurons [26, 56–62] although kinesin-1 is predominantly described for transport of APP while BDNF transport is also known to depend on kinesin-3 [19, 63, 64].

Furthermore, our experiments revealed a higher incidence of immobile vesicles in 5xFAD mouse derived neurons. There was no similar change in vesicle motility in wild-type C57BL/6 neurons which were cultured in medium conditioned by wild-type neurons transfected with hAPP-YFP or in hippocampal neurons which were treated with amyloid-beta (cf. Supplementary Figure 6 and Figure 4; see also [65]). Therefore, the change in fraction of immobile vesicles may be dependent on elevated intracellular APP level rather than on extracellular amyloid-beta peptides. APP has been described to act as an adaptor protein interacting with kinesin-1 thereby connecting vesicles to axonal transport ([39, 40] but see [41]). Consequently, APP could be responsible for the transport of a small pool of BDNF-containing vesicles to defined subcellular target compartments. A direct or indirect interaction of APP with kinesin-1 could be responsible for such a detachment of moving vesicles [66–68]. Nevertheless, the low degree of APP and BDNF colocalization argues for a largely independent transport of both proteins. On the same vein, the velocity of anterogradely directed vesicles containing APP was reported to reach up to 9 *µ*m/s (mean velocity: 4,7 *µ*m/s) [26, 69, 70], whereas we observed an average speed of BDNF-containing vesicles of 1 *µ*m/s (max 4 *µ*m/s), being consistent with previous findings [20–22, 71, 72]. This comparison of vesicle velocities speaks in favour of largely independent transport of both proteins in neurons.

One of the first changes in dynamic transport of BDNF-containing vesicles we observed was the reduction of average speed during motion of retrogradely directed BDNF-containing vesicles (Figure 4). Other deficits in transport of BDNF-containing vesicles occurred only later, suggesting that distinct mechanisms are responsible for these distinguishable deficits in BDNF transport. Fast changes in retrograde transport of BDNF-containing vesicles were described to be mediated by inhibition of vesicular ATP production [73]. However, at variance with our findings in the present study, anterograde and retrograde transport of BDNF were affected on a similar time scale as well as to a similar extent after inhibiting a key step of glycolysis [73], suggesting that short-term application of amyloid-beta(1-42) does not perturb glycolysis. Furthermore, short-term treatment with amyloid-beta(1-42) has been described to rapidly induce changes in synaptic function [74, 75]. Although electrical activity can modify vesicular trafficking, these changes are not restricted to retrograde transport [76–79], again suggesting that these cellular events are not upstream of the early deficits in retrograde transport that we observe. Acute treatment with amyloid-beta is known to modulate GSK3-signalling. GSK3-signalling can modify specifically anterograde but not retrograde transport [80, 81] suggesting a different unknown mechanism to underlie the amyloid-beta induced fast changes in retrograde transport in our study, where retrograde transport is affected first.

Long-term treatment (24 h) with amyloid-beta(1-42) as well as long-term treatment with amyloid-beta(3(pE)-42) which has been discussed recently to be a potential player in triggering Alzheimer's disease [82] affected anterograde as well as retrograde transport of BDNF (Figure 4). Similar changes were observed in hippocampal cultures of 5xFAD transgenic animals (Figure 1). 5xFAD mice produce very high levels of amyloid-beta(1-42) as well as amyloid-beta(3(pE)-42) that rapidly accumulate intraneuronally starting at the age of 1.5 months, while cognitive impairments are obvious at 4-5 months [32, 42]. Therefore, the observed deficits in transport of BDNF which occurred shortly after initiation of transgene expression in our experiments are likely to take place very early in development of 5xFAD mice and might permanently impair brain functions. Long-term treatment with amyloid-beta peptides has been described to induce several changes (for recent reviews see, e.g., [74, 83, 84]), including effects of amyloid-beta(1-42) on synaptic cargo transport [23, 65, 71, 72, 85]. Transport of synaptic cargoes is influenced by amyloid-beta oligomers in a NMDAR- and GSK-3-dependent fashion [65, 71, 72]. While Tang and colleagues [65] described an amyloid-beta induced NMDAR and GSK-3-dependent mechanism which affects velocity of synaptic cargo vesicles, Silverman's group uncovered an amyloid-beta induced NMDAR and GSK-3-dependent mechanism completely disrupting vesicle trafficking [71, 72]. A similar disruption of BDNF transport after amyloid-beta treatment could not be confirmed for the investigation period analyzed in our study (Figure 4(b)). In the 3 different assays which we used to analyze BDNF transport, we did not observe such a drastic reduction in the fraction of mobile vesicles. In our measurements, the moving or mobile vesicles were significantly slower after acute amyloid-beta(1-42) treatment or after 24 h treatment with amyloid-beta(1-42), respectively, but they were still moving. We did not observe disruption of BDNF transport and thereby increase in immobile vesicle pool after amyloid-beta treatment at all. The small but significant increase in immobile vesicle pool which we observed was only evident when hippocampal neurons overexpressed APP suggesting a function of APP and not amyloid-beta in detaching mobile vesicles from microtubules. Culturing conditions but also the way of analysis (manual versus automated tracking, e.g., [86]) could account for these different results. Another amyloid-beta induced mechanism being responsible for delayed deficits in transport of endocytosed BDNF/TrkB signaling endosomes was described by Poon and colleagues [22]. Long-term treatment with amyloid-beta oligomers decreased cell body accumulation of extracellularly applied BDNF-GFP [23] suggesting impaired retrograde transport of endocytosed BDNF/TrkB-complex. The amyloid-beta induced deficits in retrograde transport of these signaling endosomes were mediated by an ubiquitin C-terminal hydrolase dependent mechanism [22]. An additional influence of ubiquitination pathways on anterograde transport seems to be likely [87] so that also ubiquitination pathways may be upstream of the late deficits in transport deficits that we observe. Furthermore, amyloid-beta treatment has been described to induce generation of ROS [88, 89] which downregulate histone deacetylation by inhibiting histone deacetylase [90]. Deacetylation of microtubules was described to destabilize microtubule networks thereby leading to an increased number of stopping events or prolonged dwell time [60], which is in line with our observations (Figures 1 and 4).

Activity-dependent release of BDNF depends on ATP production, Calcium influx, and efficiency of glutamatergic synaptic transmission (reviewed in [91]). In addition, it is conceivable that BDNF release is also limited by the availability of BDNF vesicles ready for exocytosis at release sites. Interestingly, although the transport of BDNF was affected by amyloid-beta application as well as by transgenic expression on different levels, the release of BDNF was similar in hippocampal neurons of 5xFAD mice as compared to their control littermates. Thus, although the protein APP and the proteolytic cleavage products of APP have been shown to affect neuronal function on different levels, the depolarization induced release of BDNF was unchanged in the investigation period covered by our experiments. Recently, the role of intracellular amyloid-beta oligomers in synaptic vesicle release was investigated [92, 93]. Yang and colleagues showed that amyloid-beta monomers as well as oligomers which accumulated intraneuronally directly bind to intracellular syntaxin 1A. Furthermore, this cytosolic interaction of amyloid-beta oligomers significantly inhibited SNARE-mediated exocytosis. In contrast, we did not observe any influence on BDNF release in hippocampal neurons under these conditions. One possible explanation for this opposing result might be the importance of different protein isoforms in the release process of neurotransmitter and neuropeptide vesicles. In general, release of neurotransmitters and release of neuropeptides share similar mechanism. However, syntaxin 1A is known to be important for presynaptic exocytosis of neurotransmitter in neurons. Furthermore, it is known that syntaxin 1B and not syntaxin 1A plays an important role in release of BDNF in glial cells. Whether the identical protein machinery is important for BDNF release in neuronal cells is unknown until now. Besides the different protein isoforms, intraneuronal accumulation of amyloid-beta oligomers which is a prerequisite for direct interaction of amyloid-beta and intracellular domain of syntaxin might be a mechanism which follows deficits in transport processes. Intraneuronal accumulation of amyloid-beta(1-42) has been reported in the 5xFAD mouse model starting at 1.5 months of age. However, the transgene expression is under the regulation of the Thy1 promoter in 5xFAD mice starting with expression of mutated APP and presenilin within the first days after birth. This speaks for a delayed intraneuronal accumulation of amyloid-beta oligomers. In our hippocampal cultures derived from newborn 5xFAD mice we cannot exclude intraneuronal accumulation of amyloid-beta at this early time point. Nevertheless, we observed an effect of exogenous amyloid-beta(1-42) on transport kinetics of BDNF-containing granules within minutes. For intracellular accumulation, endocytosis of amyloid-beta and leakage of endocytosed amyloid-beta out of endosome or lysosomes are necessary, which makes it unlikely that intraneuronal accumulation of exogenous amyloid-beta is important for the fast effect on BDNF transport we observed. Furthermore, the observation that BDNF release is unaffected in 5xFAD cultures might give a hint that deficits in transport occur before intracellular accumulation of amyloid-beta. Retrograde transport deficits could influence endosomal or lysosomal membrane trafficking. These transport deficits may end in a lysosomal leakage [94] thereby resulting in an intraneuronal accumulation of Abeta and an ongoing of pathophysiological processes.

Our study reveals that the neurotrophic factor BDNF shows modest colocalization with the protein APP in vesicular structures of hippocampal neurons. The anterograde as well as retrograde transport of BDNF-containing vesicles was impaired in different model systems of Alzheimer's disease. Interestingly, short-term treatment of hippocampal neurons with amyloid-beta(1-42) resulted in a reduced average speed of BDNF-containing vesicles already after acute application. Since retrograde transport was affected prior to the anterograde direction, this suggests different mechanisms being at work for the direction-specific deficits in BDNF transport.

## 5. Conclusion

The neurotrophin brain derived neurotrophic factor (BDNF) is an important growth factor in the central nervous system. Deficits in transport of this secretory protein could underlie neurodegenerative diseases. We discovered that extracellular cleavage products of APP induced rapid changes in transport kinetics of BDNF-containing vesicles. Our data furthermore suggest a possible role of APP on detachment of moving vesicles from microtubules thereby reducing the number of mobile vesicles while release of BDNF was unaffected by transgenic expression of mutated APP.

## Supplementary Material

Supplementary Figure 1 shows the characterization of 5xFAD hippocampal cultures. Supplementary Figure 2 displays a schematic illustration of transport dynamics. Supplementary Figure 3 shows some motional properties of anterogradely and retrogradely directed BDNF-containing vesicles after hAPP expression. In figure 4, it is shown that the level of APP expression in individual cells was not correlated with the degree of BDNF-transport deficits. Colocalisation of BDNF-containing vesicles with APP is depicted in Supplementary Figure 5. Supplementary Figure 6 shows that soluble factors released into the extracellular medium disturbed anterograde transport of BDNF vesicles. Acute treatment with amyloid-beta(1-42) had no effect on transport characteristics of anterogradely directed BDNF-containing vesicles which is shown in Supplementary Figure 7 .
