# Supplementary material for: Amyloid-Beta Induced Changes in Vesicular Transport of BDNF in Hippocampal Neurons
Source: Neural Plast. 2016 Jan 10;2016:4145708. doi: 10.1155/2016/4145708 (PMC4736975; doi:10.1155/2016/4145708)
Supplement: Supplementary file 1 — Supplementary Figure 1 shows the characterization of 5xFAD hippocampal cultures. Supplementary Figure 2 displays a schematic illustration of transport dynamics. Supplementary Figure 3 shows some motional properties of anterogradely and retrogradely directed BDNF-containing vesicles after hAPP expression. In figure 4, it is shown that the level of APP expression in individual cells was not correlated with the degree of BDNF-transport deficits. Colocalisation of BDNF-containing vesicles with APP is depicted in Supplementary Figure 5. Supplementary Figure 6 shows that soluble factors released into the extracellular medium disturbed anterograde transport of BDNF vesicles. Acute treatment with amyloid-beta(1-42) had no effect on transport characteristics of anterogradely directed BDNF-containing vesicles which is shown in Supplementary Figure 7 . [file 4145708.f1.pdf]

Supplementary Figure 1

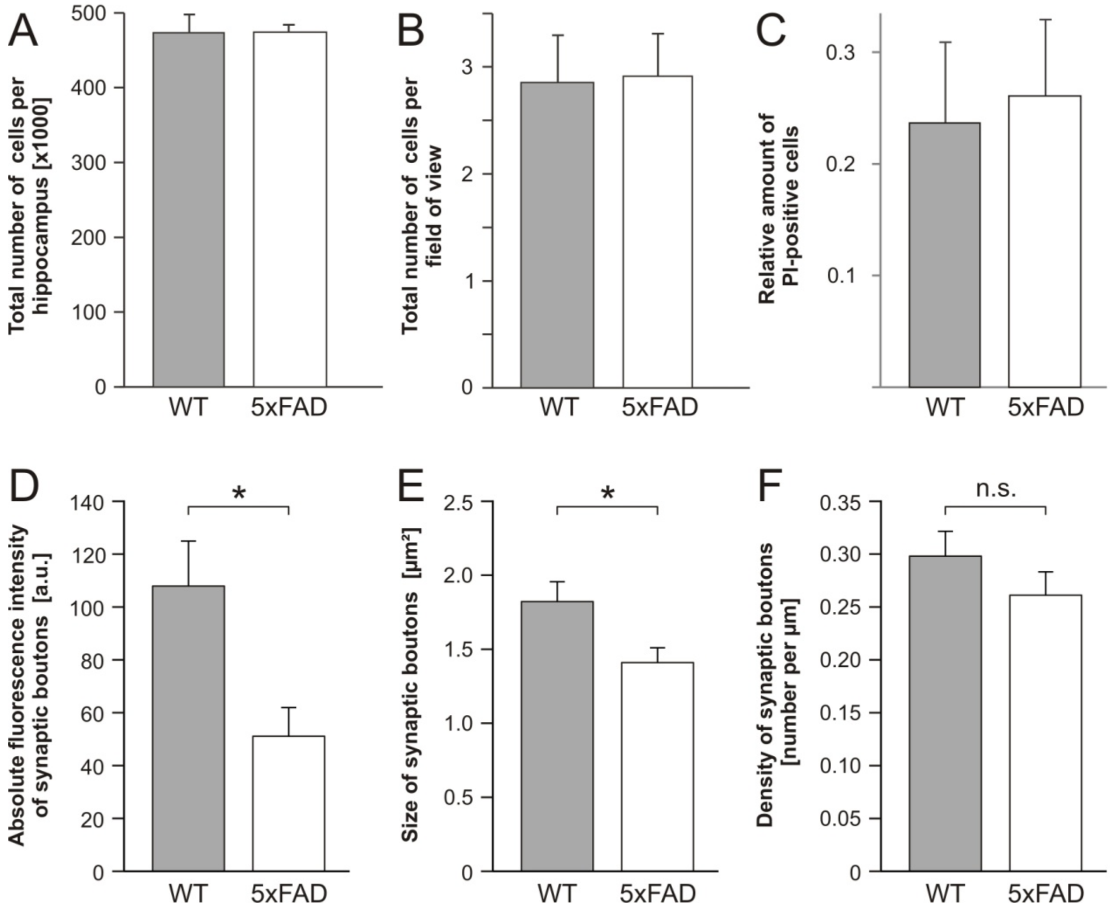

Supplementary Figure 1 Characterization of 5xFAD derived hippocampal cultures. A-B) Average total numbers of cells per hippocampus as well as average total numbers of cells per field of view were similar in 5xFAD derived cultures and cultures derived from wild type littermates. C) Fraction of Propidium iodide (PI)-positive cells were similar in both hippocampal cultures. C-E) FM-staining of hippocampal cultures revealed significant reduction in absolute fluorescence intensity of synaptic boutons and significant reduction in size of synaptic boutons in hippocampal neurons derived from 5xFAD mice. Density of synaptic boutons stained with FM dye was similar under both conditions.

Supplementary Figure 2

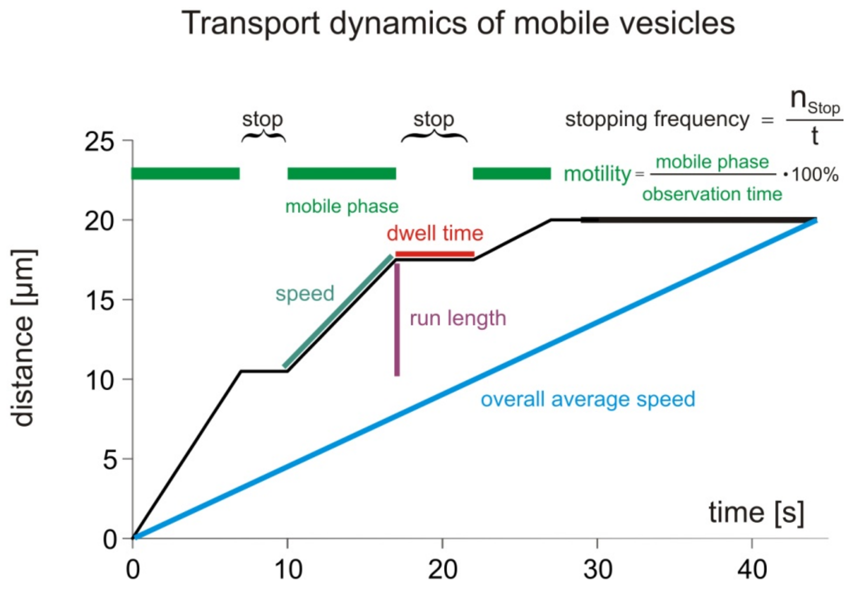

Supplementary Figure 2 Schematic illustration of transport dynamics. The movement of the vesicles was characterized by a stop and go behavior rather than constant speed. Therefore, different motional properties of mobile vesicles were analyzed. The stopping frequency quantified by the number of stops per minute and the dwell time defined by the time the vesicles spent in the same position during one stop characterize the stopping phase of the vesicles. The percentage of time while a vesicle is moving during observation defines the motility of this vesicle. The covered distance of vesicles between two stops was described by the run length of the vesicles. During the mobile phase the vesicles move with a certain speed which is described by the average speed of the vesicle during motion without considering the stopping. The average speed during motion differs from the overall average speed of BDNF-containing vesicles which was defined as the ratio of distance covered to observation time.

Supplementary Figure 3

	day	Anterograde transport in hAPP-expressing neurons				Retrograde transport in hAPP-expressing neurons			
		stopping frequency [1/min]	dwell time [s]	run length [µm]	motility [%]	stopping frequency [1/min]	dwell time [s]	run length [µm]	motility [%]
Ctrl	1	9.63 ± 0.64	1.23 ± 0.09	14.53 ± 1.75	75.1 ± 2.2	13.79 ± 0.62	1.69 ± 0.14	5.90 ± 0.96	61.9 ± 2.3
	2	11.15 ± 0.58	1.36 ± 0.11	13.17 ± 2.08	71.7 ± 2.1	13.11 ± 0.60	1.66 ± 0.10	6.44 ± 0.96	61.2 ± 2.3
	3	10.2 ± 0.65	1.2 ± 0.08	15.24 ± 1.90	73.9 ± 2.2	14.24 ± 0.57	1.49 ± 0.07	5.25 ± 0.73	62.7 ± 2.1
	Total	10.37 ± 0.36	1.26 ± 0.06	14.29 ± 1.12	73.5 ± 1.3	13.71 ± 0.35	1.62 ± 0.06	5.87 ± 0.52	61.9 ± 1.3
hAPP	1	10.67 ± 0.74	1.35 ± 0.12	14.83 ± 2.20	73.5 ± 2.2	13.87 ± 0.61	1.64 ± 0.10	5.12 ± 0.89	61.0 ± 2.0
	2	11.05 ± 0.74	1.10 ± 0.07	13.41 ± 2.40	76.1 ± 2.4	15.81 ± 0.57	1.41 ± 0.08	2.86 ± 0.31	62.7 ± 2.2
	3	11.89 ± 0.64	1.41 ± 0.11	9.09 ± 1.39	69.4 ± 2.3	15.46 ± 0.56	1.76 ± 0.13	4.54 ± 1.07	58.5 ± 1.9
	Total	11.23 ± 0.41	1.31 ± 0.06	12.30 ± 1.15	72.7 ± 1.3	14.96 ± 0.34	1.63 ± 0.07	4.34 ± 0.53	60.5 ± 1.2

Supplementary Figure 3 Expression of hAPP reduced vesicular transport of BDNF vesicles. Dissociated hippocampal neurons of C57BL/6 mice were cotransfected at 10 DIV with BDNF-mCherry and hAPP-YFP. The dynamic behavior of BDNF-containing vesicles was analyzed by live cell imaging 1-3 days after transfection. Table shows the motional properties of anterogradely and retrogradely directed BDNF-containing vesicles which do not show any significant differences throughout the first days after hAPP expression.

Supplementray Figure 4

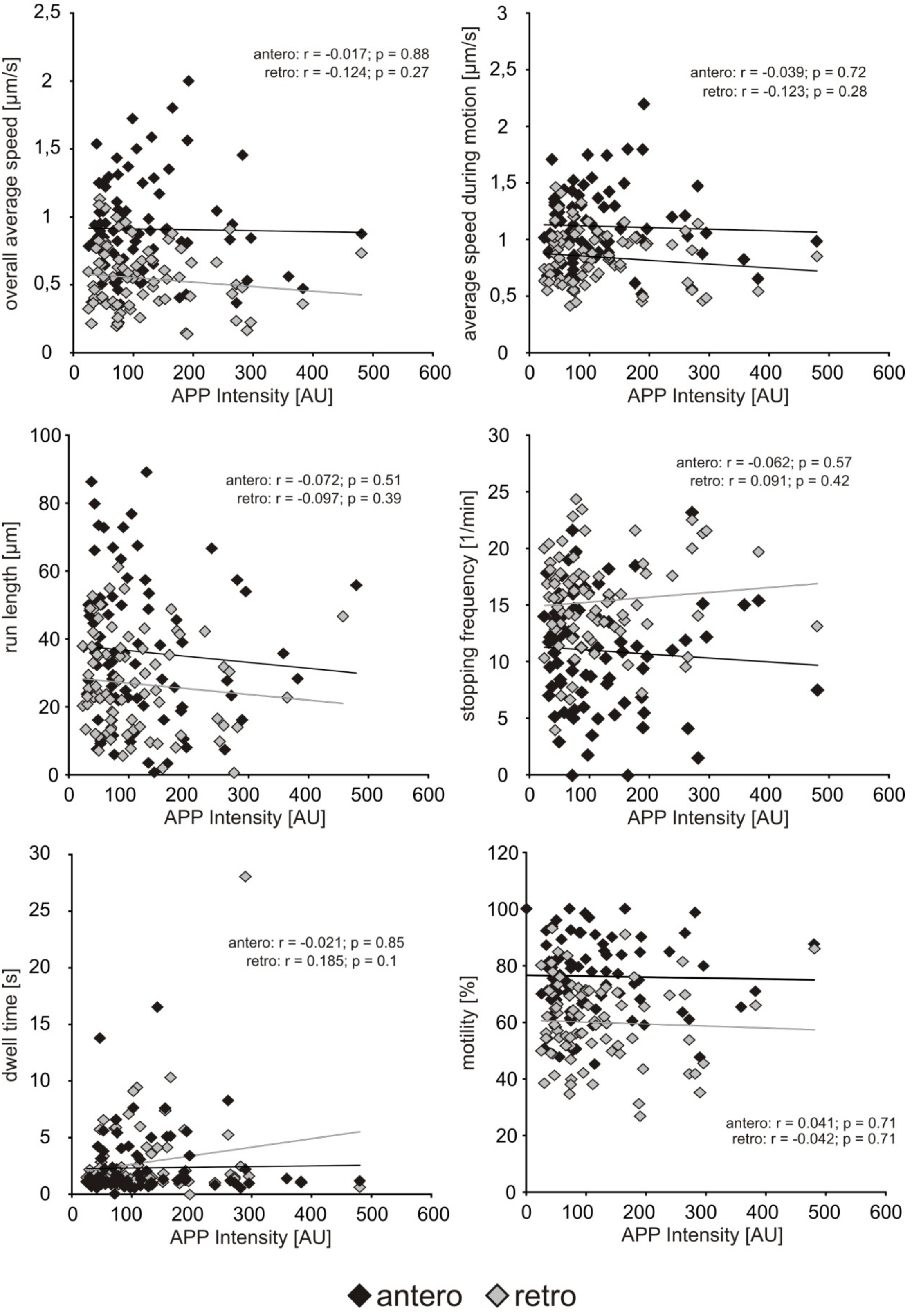

Supplementary Figure 4 The level of APP expression in individual cells showed no correlation with the degree of BDNF-transport deficits. Dissociated hippocampal neurons of C57BL/6 mice were cotransfected at 10 DIV with BDNF-mCherry and hAPP-YFP. Values of the respective motional properties of BDNF-containing vesicles (determined at day 1-3) were plotted against fluorescence intensity of somatic APP-YFP. Note, that there was no correlation between APP level and motional properties of individual cells. (Pearson correlation coefficient (r) < 0.1; p-value > 0.5).

Supplementary Figure 5

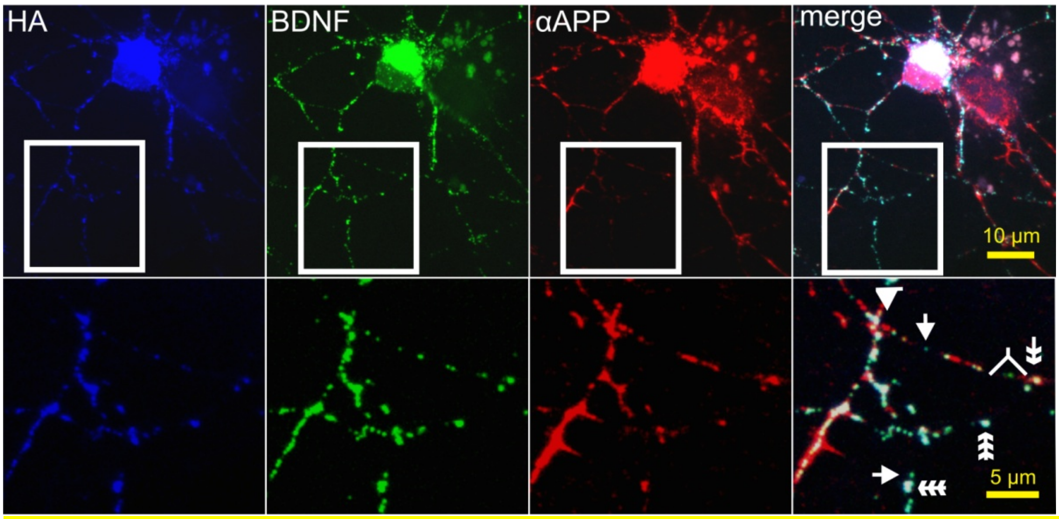

Supplementary Figure 5 A subpopulation of BDNF-containing vesicles colocalizes with APP. A) Dissociated hippocampal neurons of 5xFAD animals were transfected with N-terminal HA-tagged proBDNF-GFP and were double-immunostained with an antibody directed against the HA-tag (blue) and with an antibody directed against the N-terminus of endogenous APP (red). The C-terminus of BDNF is fluorescently labeled with GFP (green). (Upper part) Representative picture showing a transfected and immunostained hippocampal neuron. (Lower part) Higher magnification of boxed area in upper panel. Mature BDNF colocalized with the HA-tagged prodomain of BDNF and APP in vesicular structures (triple arrowhead). In addition, BDNF colocalized with the N-terminal part of APP (double arrowhead) or with the prodomain of BDNF (single arrowhead) respectively.

Supplementary Figure 6

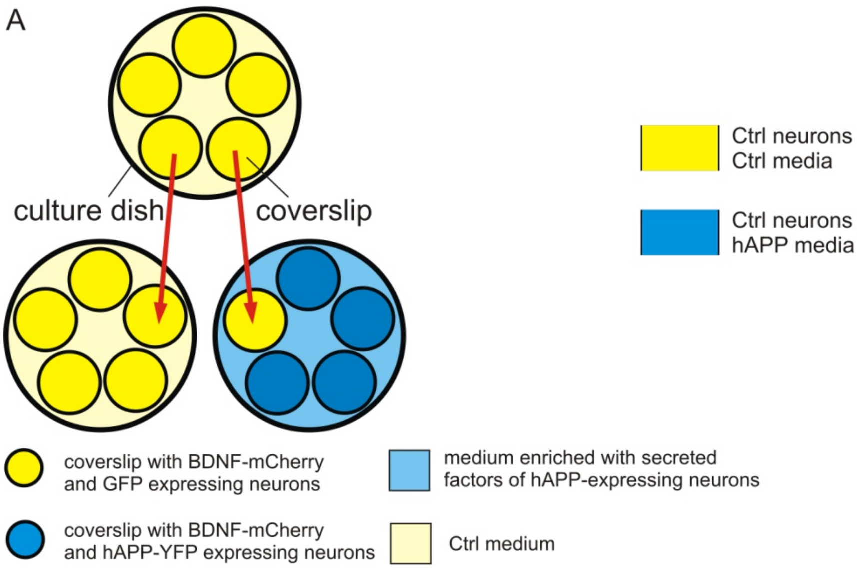

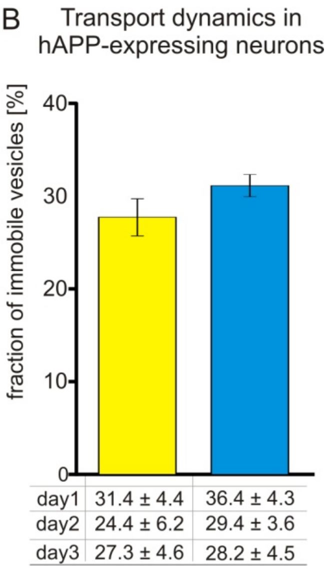

C

anterograde	day	overall average speed [μm/s]	average speed during motion [μm/s]	run length [μm]	stopping frequency [1/min]	dwell time [s]	motility [%]
Ctrl neurons in Ctrl media	1	0.98 ± 0.09	1.19 ± 0.07	15.06 ± 3.33	13.41 ± 2.16	1.12 ± 0.12	75.1 ± 3.4
	2	1.12 ± 0.11	1.36 ± 0.09	21.26 ± 5.13	10.71 ± 1.09	1.31 ± 0.18	73.7 ± 3.7
	3	1.07 ± 0.09	1.23 ± 0.07	21.58 ± 4.37***	9.59 ± 0.99	0.96 ± 0.09	79.9 ± 3.0
	Total	1.06 ± 0.05***	1.26 ± 0.05***	19.42 ± 2.53***	11.17 ± 0.85	1.13 ± 0.08	76.3 ± 2.0
Ctrl neurons in hAPP media	1	0.90 ± 0.07	1.14 ± 0.06	8.91 ± 1.66	11.46 ± 0.78	1.23 ± 0.12	73.4 ± 3.0
	2	0.86 ± 0.07	1.09 ± 0.06	11.70 ± 2.54	12.21 ± 0.93	1.24 ± 0.09	70.4 ± 3.2
	3	0.74 ± 0.05	0.97 ± 0.05	6.55 ± 1.22***	14.26 ± 0.75	1.16 ± 0.07	70.2 ± 2.3
	Total	0.82 ± 0.04***	1.05 ± 0.03***	8.77 ± 1.04***	12.84 ± 0.48	1.2 ± 0.05	71.2 ± 1.6

Supplementary Figure 6 Soluble factors released into the extracellular medium disturbed anterograde transport of BDNF vesicles. Dissociated hippocampal neurons of C57BL/6 mice were transfected at 10 DIV with BDNF-mCherry and a control plasmid or hAPP-YFP, respectively. Cover slips with control neurons were transferred after transfection into culture dish with hippocampal neurons transfected with hAPP or control plasmid, respectively. The dynamic behavior of BDNF-containing vesicles was analyzed by live cell imaging 1-3 days after transfection. A, Schematic illustration of experimental setup. B, Bar diagram indicates the mean percentage of immobile BDNF-containing vesicles under ctrl condition and in the neighborhood of hAPP expressing neurons. C, Table shows the mean value of indicated motional properties of anterogradely directed BDNF-containing vesicles during the whole observation time (total: day 1-3 after transfection) or during the respective observation day. (*** $p < 0.001$; two-way ANOVA; n > 34 vesicles per day, group and direction; 109 neurons, 6 preparations).

Supplementary Figure 7

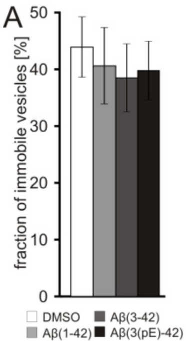

B

	overall average speed [μm/s]	average speed during motion [μm/s]	run length [μm]	stopping frequency [1/min]	dwell time [s]	motility [%]
DMSO	0.94 ± 0.1	1.21 ± 0.08	12.20 ± 3.70	11.04 ± 1.16	1.29 ± 0.20	73.2 ± 4.0
Aβ(1-42)	0.73 ± 0.07	0.95 ± 0.06	7.86 ± 2.04	13.45 ± 1.16	1.14 ± 0.11	71.5 ± 3.4
Aβ(3-42)	0.98 ± 0.09	1.16 ± 0.07	16.62 ± 3.37	10.47 ± 1.20	1.06 ± 0.10	77.9 ± 3.4
Aβ(3(pE)-42)	0.82 ± 0.09	1.05 ± 0.07	12.22 ± 3.94	12.34 ± 1.04	1.28 ± 0.12	70.9 ± 3.4

Supplementary Figure 7 Acute applications of amyloid-beta(1-42) altered retrograde BDNF-transport. A) Bar diagram shows the percentage of immobile BDNF-containing vesicles in hippocampal neurons after acute treatment with different amyloid-beta peptides ($p > 0.5$; one-way ANOVA; $n > 13$ neurons per group, 4 preparations). B) Acute treatment with amyloid-beta(1-42) had no effect on transport characteristics of anterogradely directed BDNF-containing vesicles. The transport characteristics were similar under all conditions here. There was no statistical difference after treatment with different amyloid-beta peptides.
